# The increasing relevance of immunobiology within a connected animal science curriculum

**DOI:** 10.1093/tas/txad007

**Published:** 2023-01-14

**Authors:** Kieran G Meade

**Affiliations:** School of Agriculture and Food Science, University College Dublin, Belfield, Dublin 4, Ireland; Conway Institute of Biomolecular and Biomedical Science, University College Dublin, Belfield, Dublin 4, Ireland; Institute of Food and Health, University College Dublin, Belfield, Dublin 4, Ireland

**Keywords:** animal science, livestock immunity, one health, threshold concepts

## Abstract

Modern technological agriculture emerged in the 20th century and has expanded into a global enterprise occupying approximately 38% of the Earth’s land area and accounting for over 40% of the world’s workforce. The United Nations Food and Agriculture Organization estimates that to feed a world population of 9-billion people in 2050 will require an almost doubling of overall food production, including meat, dairy, and egg production over 2010 levels. However, our collective ability to meet this demand cannot be taken for granted. Despite many successes, global agricultural systems now face multiple unprecedented challenges including a dearth of new treatments for livestock diseases. The discovery of antibiotics led to a complacency now reflected in a dependency on exogenous antimicrobials and a growing threat of antimicrobial resistance. Developments within the field of immunobiology had led to significant breakthroughs in understanding of human health and disease. However, despite over 60% of infectious diseases being zoonotic in nature and nonhuman animals acting as an important disease reservoir, research in livestock immunobiology has not been as resourced. As a direct result, recalcitrant animal diseases continue to threaten sustainability of animal production systems, security of the food chain and human health. It is within the context of collective One Health action that ambitious innovation in the connectivity of animal science undergraduate curricula is urgently required, specifically to include threshold concepts in immunobiology. Fostering transformative learning is critical to equip future generations of animal scientists with the knowledge and interdisciplinary skills to counter these existential challenges of our time.

## INTRODUCTION—A RENEWED FOCUS ON DEVELOPING AND MAINTAINING HEALTH IS ESSENTIAL TO LIVESTOCK PRODUCTION SYSTEM SUSTAINABILITY

The practice of agriculture is characterized by continual innovation to overcome challenges inherent in the different farming enterprises and to survive in a highly competitive, low-margin marketplace. This constant quest for efficiency, savings, and new solutions is also reflected across research programmes and in the urgency of stakeholders to improve transition of research outputs into practice in industry and on farms. While it might be argued that the core principles underpinning animal production enterprises have not radically changed since the advent of formalized animal science education and are therefore as relevant now as ever to educational practice, unprecedented recent pressures mean that many aspects of animal science are undergoing a process of fundamental change (Ruben et al., 2021). The technological revolution that is underway in agriculture means that graduates need to be prepared for these fundamental shifts in livestock production systems to develop and support the industry response to unprecedented pressure to feed the world but also to play a more active role in tackling the increasingly critical issue of the planet’s sustainability (Nicol, 2021).

Agriculture connects a global community of practitioners and educators, and yet overcoming key limitations of optimal animal production remains a challenge—specifically the recalcitrant issues of animal disease which threaten overall system sustainability (Perry et al., 2018). Animal diseases reduce annual production by around 20%, lead to an overreliance on antibiotics and have negative animal welfare implications (Dupjan and Dawkins, 2022). Estimates suggest that US$ 300 billion a year is lost annually to animal disease (IFAH, 2012), a figure which is widely recognized as unsustainable at farm, national, and global levels. Improving the lifetime performance of farm animals is key to more sustainable livestock systems. The importance of animal health to food supply security is emphasized when it is considered that eight animal species supply 90% of the world’s food. It is now widely recognized, particularly in light of the recent Covid-19 pandemic, that a paradigm shift is required in our approach to all livestock diseases, particularly when one considers the related risks associated with antimicrobial resistance (**AMR**) and zoonotic diseases (Torres-Velez et al., 2019). More than 60% of human infectious diseases are caused by pathogens shared with wild or domestic animals (Taylor et al., 2001). However infectious diseases do not just impact human and animal health but also on the economic sustainability of agricultural systems and rural economies. All these issues affect consumer attitudes to animal production and control of disease can be societally divisive. There are also manifest lessons for us all in how to reduce the risks associated with future zoonoses from our ongoing Covid-19 experience, and there is a critical urgency to engender a paradigm shift embedded at a pedagogical level in undergraduate animal science curricula.

Recognition of the need to modernize the animal science curriculum is not new and R.G. Kauffman previously referred to the curriculum as a “moving target” (Kauffman, 1992) due to the changing needs of both students and society. Acknowledgment of the magnitude of the challenges facing the agriculturalists of the future has led to a number of recent analyses seeking ways to ensure graduates of animal science are endowed with the knowledge and skills to meet the diverse, complex, and changing challenges in animal agriculture (Mayberry et al., 2021). Kauffman recognized the essential need for the curriculum to change as pedagogy that does not meet the needs of changing world will ultimately fail (Kauffman, 1992). It is also understood that the key to the provision of the industry with knowledge-leaders of the future is also innovation in terms of pedagogy (Buchanan, 2008). A national scoping exercise on animal science undergraduate education was recently conducted across 49 academic institutions in the USA (Erickson et al., 2020). The survey identified a clear demand for more practical, collaborative, and computational assignments which included research experience at undergraduate level; and to “catalyze knowledge sharing across disciplines, institutions, and experiences.”

Immunobiology is the branch of biology that deals with the biological effects of the immune system, with an important focus on integration and the development and maintenance of health, and not merely a focus on disease—a field which has “exploded” in recent times (Bottaro et al., 2021). Of relevance in terms of optimizing immune system development in livestock to build resilience, immunobiology is also directly relevant to specific diseases which seriously impact global animal production efficiency including bovine tuberculosis, mastitis, avian and porcine influenza, and African Swine Fever to name but a few. Controlling infections at source and prevention of disease spread is a critical niche within which the farmer and animal scientist play pivotal roles.

This study seeks to define the core foundational concepts required to promote a paradigm shift in the undergraduate animal science curriculum based on a comprehensive needs assessment from the student, university, employer, and societal perspectives.

## THRESHOLD CONCEPTS IN IMMUNOBIOLOGY FOR TRANSFORMATIONAL LEARNING IN ANIMAL SCIENCE

Introduced by Jan Meyer and Ray Land, a “Threshold Concept” is a concept that, once understood, changes the way that a person thinks about a topic (Meyer and Land, 2003, 2006). Threshold concepts have several attributes or characteristics that ultimately result in a reformulation of the learner’s frame of meaning and are specifically listed as: “Transformative,” “Irreversible,” “Integrative,” “Bounded,” and “Troublesome.” Transformative refers to the manner by which a threshold concept will open up a new and previously inaccessible way of thinking about a topic; irreversible in the sense that once understood, a threshold concept cannot be viewed in another way; integrative in terms of bring previously unappreciated aspects of the subject together; bounded by serving a specific purpose; and troublesome as it may cause some discomfort as the student is forced to depart from old approaches or previous ways of thinking about a subject (For recent review: see Stopford, 2021).

Threshold concepts can be viewed as a portal to new ways of thinking—concepts that are necessary for a student to progress in their learning, to bring fresh perspectives to problem-solving and to engage more effectively in shaping their future careers. The most appropriate threshold concepts in immunobiology of relevance to undergraduate animal science students will emerge from a detailed needs analysis from a student, university, employer, and societal perspective (Table 1).

**Table 1.** Needs analysis conducted to define relevant threshold concepts

Group	Need
Student	To visualize the extensive role of animal production systems in meeting the current demand for animal protein globally and to understand how this is likely to change going forward.
	To envisage the potential consequences of increasing livestock numbers on antimicrobial usage and the challenges of disease prevention.
	To grasp the interconnected nature of immunobiology and use integrated curriculum knowledge to support optimal animal health on farms.
	To communicate best practices on farming practices to maintain biosecurity and prevent disease.
	To engage with emerging research, new technologies and other stakeholders to improve antimicrobial stewardship and design new solutions for livestock disease.
	To advocate for the critical role of farmers as guardians of animal welfare, in the protection of the food chain and as protectors of human health.
	To appreciate the urgency of controlling diseases at source from a farm sustainability and human health perspective.
	To become ambassadors for agriculture and engage in evidence-based design of new solutions for animal diseases.
University	To identify areas of economic growth where animal scientists are currently underrepresented.
	To equip future animal science students with the knowledge and skills to participate in the animal health economy.
	To develop routes for animal science undergraduates into further education and research.
	To enhance graduates’ ability to critically assess research findings of relevance.
Employer	Animal science graduates with relevant knowledge to pursue new solutions for animal diseases.
	Animal science graduates with expertise in technology.
	Animal science graduates with comprehensive ability to grow opportunities in new scientific areas.
Societal	To recognize the importance of supporting primary producers of food as a societal good to protect human health.
	To support initiatives including the introduction of new methods and technologies to prevention of disease at source.
	To understand the interconnected nature of the global ecosystem and thereby engage in the promotion of One Health solutions.

Threshold concepts are a pedagogical tool that informs curriculum design in order to achieve transformational learning. Transformational learning is defined as the process of “deep, constructive, and meaningful learning that goes beyond simple knowledge acquisition and supports critical ways in which learners consciously make meaning of their lives” (Simsek, 2012). From a theoretical perspective, Batzli et al. outline how the threshold concept of “variation” might influence students’ understanding of core concepts including genetics and evolution in biology (Batzli et al., 2016), as an example. The authors outline a scheme to allow students experience and apply the idea of variation in such a way that it transforms their future understanding and learning and as an integrative bridge between concepts and competencies.

Immunobiology is not a standard part of the curriculum in many animal science undergraduate courses and therefore threshold concepts have critical relevance to achieving deeper understanding in this complex area. Several aspects of learning that can be instilled as transformative, in the sense that it will help unravel the complexity of infectious diseases that are so relevant in the practice and careers of all animal scientists. Once new connections between diseases and fundamental concepts (e.g., evolutionary biology) are established, students will start to see the world of health and disease in a new, irreversible light. It is a critical objective of the use of these threshold concepts to integrate immunobiology into other aspects of the education, career, and life of the animal scientist which will empower them to contribute intellectually as well as practically in their future. Overcoming the troublesome discomfort associated with the complexity inherent in immunobiology will enable them to solve unforeseen problems associated with animal disease in an informed and evidence-based manner.

**Figure 1.**
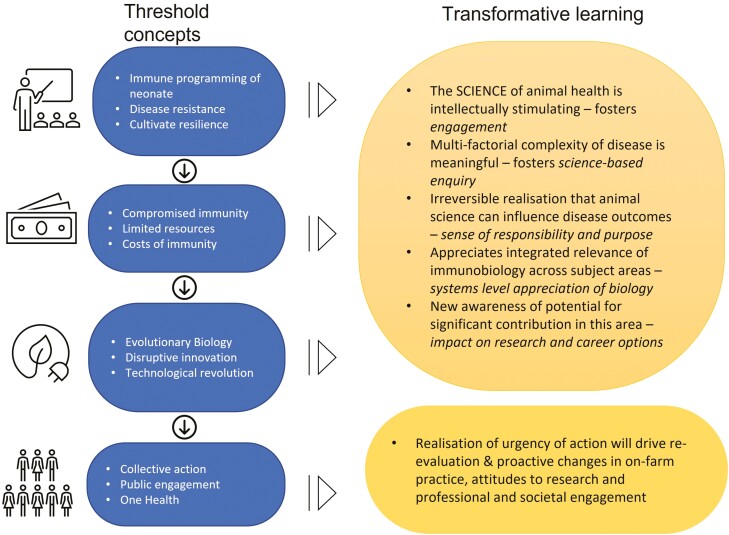
A journey through the threshold concepts in immunobiology of relevance to animal science which illustrates the resulting transformative learning.

As shown in Table 2, 13 key threshold concepts have been identified based on the needs analysis conducted here as relevant to the education and practice of animal scientists. They fulfill the criteria for threshold concepts, primarily as they may challenge long-standing views in relation to animal health and disease. They are transformative in the way they will precipitate the emergence of a new responsibility for the promotion of animal health in animal scientists. Rather than a reactive attitude to controlling disease once it has appeared in livestock, the threshold concepts will help promote proactive engagement in disease prevention. It is critical that these transformative concepts tackle the perceived complexity around immunobiology and as such, they will become a portal to enhanced understanding, foster a sense of control over complex animal diseases and yield an irreversible realization that animal scientists have a key role to play as farmers and as scientists in the design, adoption, and ultimate success of new animal health solutions (Figure 1).

**Table 2.** Threshold concepts in immunobiology from pedagogical and societal perspectives

Threshold concept		Relevance to animal science education and practice
1	Immune programming	Programming for optimal animal health begins prior to conception.
2	Disease resistance	Multiple factors determine resistance against disease and resistance is dynamic.
3	Cultivate resilience	Resilience against diseases can be cultivated—and this requires an understanding of immunobiology.
4	Compromised immunity	Compromised host immunity can result from multiple cumulative stressors exceeding the physiological reserve and will expose the individual to multiple, repeated and often synchronous opportunistic infections.
5	Limited resources	Domestication and selection pressures have altered animal homeostasis and homeorhetic function.
6	Costs of immunity	Quite apart from the unsustainable economic costs of disease, immunobiology underpins successful capture of all other traits of agricultural interest.
7	Unprecedented challenges	There are multiple downsides to antibiotic dependency. The advent of AMR and zoonotic infections mean business as usual is no longer an option.
8	Evolutionary biology	Comparative evolutionary biology holds clues to overcoming many infectious diseases.
9	Disruptive innovation	New solutions for animal diseases are urgently required and future agricultural sustainability depends on disruptive innovation in immunobiology (e.g., immunotherapeutics). Knowledge really is power in terms of disease control.
10	Technological revolution	Combining that science-based foundation with biosensors and other biological (e.g., gene-editing) and computational technologies will revolutionize animal health. New and expanded career opportunities for future animal scientists.
		Relevance to societal engagement and protection
11	Collective action	Animal health is everyone’s business—no profession is an island, and collective action is needed to overcome these existential challenges. Farmer commitment is key to reduction in antimicrobial usage and adoption of new solutions.
12	Public engagement	An informed public is key to societal acceptance of evidence based scientific and technological solutions. Societal involvement will also improve participation, engagement, foster a sense of control and ownership and reduce societal division.
13	One Health	Animal health underpins agricultural sustainability and is critical for the safety of the food chain and human health. Ultimately, a One Health focus is critical to the future of the planet and our species.

### Approaches and Tools for Teaching Immunobiology “Threshold Concepts” of Relevance to Animal Science

Inherent in the fundamental integrative nature of immunobiology is a risk to creating a barrier to effective learning via the inability to make coherent links in the mind of the student and thereby fail to achieve the intended aim of transformational learning. In this regard, the teacher has a critical role to play, particularly in the selection of the most appropriate learning tools to support transformational learning. A principal key motivation for incorporating threshold concepts in immunobiology into the undergraduate animal science curriculum is to reduce the “fear factor” around the perceived complexity underlying the multifactorial nature of animal diseases (Zeyer et al., 2017). It is therefore important that students realize that discomfort and vulnerability are important steps in authentic transformational learning (Robinson, 2021). Some suggested approaches which are likely to promote engagement with threshold concepts and enhance transformational learning are discussed below.

#### Immunobiology—“the seeing”

##### Active or experiential learning:

Active learning involves moving the focus away from the instructor toward the student and has shown important relevance in agriculture curricula previously (Quarrie, 2014). It is critically important to achieve engagement by students (Barnett et al., 2005) which will dismantle misappropriated perceptions in immunobiology and prepare the students for transformative learning. Students must do more than just listen but engage in solving problems, and there is a significant opportunity to build a strong curriculum relevance between the more basic concepts in immunobiology through direct linkages with common livestock diseases. Experiential learning can also be used to strengthen the linkage between teaching and research (Healey and Jenkins, 2007) so students identify knowledge gaps and discuss potential strategies for disease amelioration.

Active learning can be achieved via the use of technology reflected in the diversity of teaching approaches employed and through the incorporation of multimedia technology (Meyer et al., 2014). There are a number of open educational resources for teaching immunology available (Faggioni et al., 2019), and these can be incorporated into formative assessments (Brown et al., 2020). Visualization of the immune system can sometimes be a challenge but the advent of virtual reality platforms offer emerging tools for engagement in immunobiology (Faggioni et al., 2022). An interesting example may be to include the use of a virtual farm (Calsamiglia et al., 2020) which will enable students to understand the implications of decision-making on health, welfare, as well as economic outcomes of practical relevance on farm. This may become a particularly relevant tool in achieving active learning objectives in animal science students from nonfarming backgrounds. Practical classes are another valuable tool to help students to grasp the underlying scientific concepts in immunobiology because they allow students to visually see the integration of disparate physiological events. They also bring the theory to life, and allow students to experience some applications of the theory (Horrigan, 2018).

Importantly, active learning is also a critical approach in the achievement of equality, diversity, and inclusion as it has core relevance within the diverse and changing demographic profile of the modern animal science undergraduate (Theobald et al., 2020).

#### Immunobiology—“the communicating”

##### Terminology and group-based informational interviews:

A major impediment to learning in immunobiology can be the discipline-specific and expansive terminology. It is therefore important to use multiple tools to grow the students comfort level in the discussion of basic concepts which can then be used as a platform to develop more detailed discussions. The use of immunobiology crossword puzzles can be a useful mechanism to foster familiarity with terminology as well as a formative assessment tool. Engaged animal science students often have relevant questions about why progress in reducing or eradicating certain diseases has not met with success to date (e.g., bovine tuberculosis). Therefore a very useful tool to improve student communication skills and the acquisition of new knowledge is via group-based expert-engaged informational interviews (Hobson and Townsend, 2010). These can be conducted online, where the group of undergraduate students research and identify a suitable expert in the disease area (practitioner or researcher or other stakeholder), prepare interview questions, conduct the interview, and then present findings to the class. Many experts are more than willing to engage students, particularly as interviews can be conducted easily online. As recently outlined in a publication for the design of undergraduate immunology courses (Justement and Bruns, 2020), these rubrics were developed by teams of faculty and educational professionals and provide a method of assessing the developmental progression of student accomplishment, culminating in advanced and integrative learning. By implementing assessments involving presentations, the student’s progress in developing oral communication skills can be evaluated in addition to their theoretical understanding. Animal health is important from a multitude of stakeholder perspectives. Discussion groups where students adopt the perspective of different stakeholders (farmer, veterinarians, consumers, government) will help develop the multiliteracy skills that will be a valuable asset for both transformational learning and for future employment prospects.

#### Immunobiology—“the reflection”

##### Reflective practice:

 Previous reports have identified that “students struggle to express how the knowledge they have learned is meaningful, relates to their lives, or transfers into tangible skills” (DuRose and Stebleton, 2016). Therefore, it is critical that reflective practice be employed to help students articulate their learning and to support them in developing the language and communication skills to master the concepts in immunobiology and additionally to connect various aspects of the undergraduate curriculum as well as to realize the practical (and transformative) relevance of this new knowledge (Carroll, 2010). Reflection is the consideration of why things are done a particular way and asking if it could be done differently. The outcome of this reflection is to “create new understanding which in turn may lead to increasing choices, making changes, or reducing confusion” (Lyons, 2006; Carroll, 2010). Action learning including interviews formalizes the reflection process by organizing the learners into groups and challenges their underlying assumptions as they reflect on their shared experiences (Lexis et al., 2021). This is a tool previously used to teach agricultural topics (Battisti et al., 2008; Shoulders and Myers, 2013) and can be adopted for purposes in immunobiology. According to Ramsden, “learning should be seen as a qualitative change in a person’s way of seeing, experiencing, understanding, conceptualizing something in the real world” (Ramsden, 1988), and so students should be encouraged to reflect on the implications and practical relevance of the relevant theoretical concepts (Erickson et al., 2021).

### Learning Outcomes as a Result of Incorporation of Immunobiology “Threshold Concepts”

Students will acquire:

- Confidence discussing various components of the immune system.
- Clarity in relation to the relevance of immunobiology within a connected curriculum context.
- Deep understanding of immunobiology and its direct relevance to the major issues that the animal sector faces.
- The ability to apply this knowledge to meaningful assessment of current farming practices and approaches to diseases in livestock.
- An understanding of their role in the disease process and steps that both farmers and animal scientists can take toward disease prevention.
- The ability to identify current barriers to disease control and the ability to devise potential new solutions.
- A foundation in the ability to interrogate and apply emerging research.
- An understanding of technological developments that will enhance future disease control.
- A holistic appreciation of the complex interconnectedness of factors that require a One Health approach to foster system sustainability.
- An ability to respond to future disease challenges in a more prepared, evidence based and effective manner.

## IMMUNOBIOLOGY WITHIN A CONNECTED ANIMAL SCIENCE CURRICULUM

Immunobiology threshold concepts do not exist in a vacuum within the context of the overall undergraduate animal science curriculum. It is therefore critical that innovative approaches are taken not only to foster learning of the threshold concepts in immunobiology but also to embed them within a connected framework linking the curriculum, career, and societal benefits which will maximize the results achieved from transformative leaning.

### Pedagogical Innovation and the Inclusive Concept of Connectivity Within an Animal Science Curriculum

A connected curriculum sets out a plan for an integrated approach to education (Fung, 2017). As well as defining the relationship between learning and research, it also describes the connections between disciplines to provide a more holistic educational approach. It promotes interdisciplinary questions and challenges, encouraging students to critically question the nature of evidence and knowledge production across different subject fields. This is particularly critical in areas of animal health where it is often critical to challenge long-accepted dogma in the light of the wealth of evidence emerging as a result of technological advances in order to accelerate progress against recalcitrant diseases in livestock. Explicitly making the curriculum visible through improved alignment will ultimately aid in achieving the required learning outcomes (Wijngaards-de Meij and Merx, 2018). The benefits of a connected curriculum extend beyond the student and threshold-concept led research partnerships that can also be used to “open up a dialogue with the staff in a discipline” (Cousin, 2010) which would be very relevant given the incorporation of multiple new curriculum elements (Bovill and Woolmer, 2019). This integration across the curriculum promotes the dismantling of silos toward holistic and integrated systems approaches, required for future sustainable solutions in animal science.

**Figure 2.**
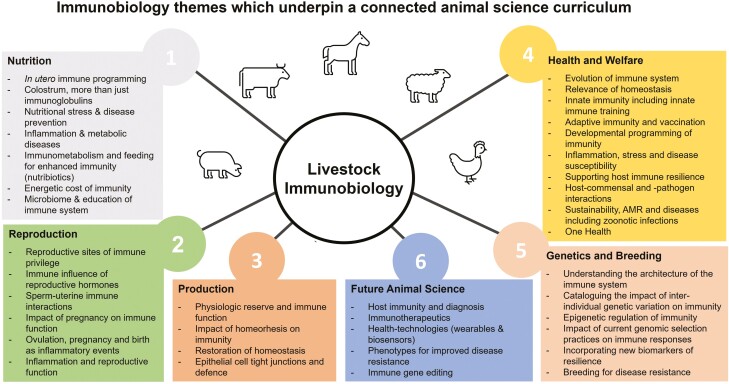
Curriculum congruence achieved by embedding immunobiology within a connected animal science curriculum.

A nonexhaustive example of how a connected curriculum could incorporate core concepts in immunobiology to achieve curriculum congruence and connectivity across the traditional animal science curriculum is shown in Figure 2. In reality, a two-way relationship between many of these discipline areas and immunobiology exists and this is one example to illustrate the cross-relevance of emerging concepts in immunobiology (Porter et al., 2021).

The undergraduate animal science student population has also continued to diversify with increasing participation rates from females, and students from nontraditional urban backgrounds (Buchanan, 2008). Therefore, no one-size-fits-all approach can be adopted in relation to curriculum design; and flexible components will undoubtedly be attractive to new entrants. There are many appealing attributes to the study of immunology as students see relevance to their own bodies and as an opportunity to engage with a physiological system that is so anatomically disseminated (Stranford et al., 2020). They also appreciate that immunology is a dynamic field in which important conceptual advances are still emerging, as recently illustrated in relation to Covid-19.

### Future Proofing the Animal Science Graduates with Immunobiology Literacy

One certainty is that livestock production systems of the future will have undergone radical changes if the seemingly incongruous challenges of feeding a growing global population while developing sustainable food systems are to be realized. The changes required across agricultural systems but particularly in animal science are not insignificant (Ruben et al., 2021). It is widely recognized that in order to achieve EU and global best practice, more active engagement in disease prevention in agriculture is required. Proactive rather than reactive approaches reduce the spread of disease, and improve treatment as well as cost outcomes, highlighting a distinct prominent role for the farmer and animal scientist. Therefore, pedagogical innovation in the animal science undergraduate curriculum, specifically in regard to immunobiology is critical to engendering these outcomes.

To reduce disease occurrence, farmers need to engage in a more informed way with allied health professionals to improve disease resilience, encourage early intervention to improve treatment outcomes and to realize the urgency of appropriate antibiotic stewardship. Farmers’ knowledge and expectations of antimicrobials have been strongly associated with usage (Kramer et al., 2017), and reducing usage is the single greatest weapon against AMR. As many animal scientists are current or future practitioners on farm; or will directly interact with farmers via advisory and sales roles, this realization should transform demand for antimicrobials from veterinary practitioners. A significant stumbling block toward solutions in agriculture is the adoption of new knowledge; and farmers’ attitude is also key to technology uptake and adoption (Liu et al., 2019). Thereby transformational learning in immunobiology will ultimately help bridge the gap between theory and practice in animal health. This will happen directly via improved farming practices but also indirectly via informed and realistic engagement with disease control and eradication measures.

**Figure 3.**
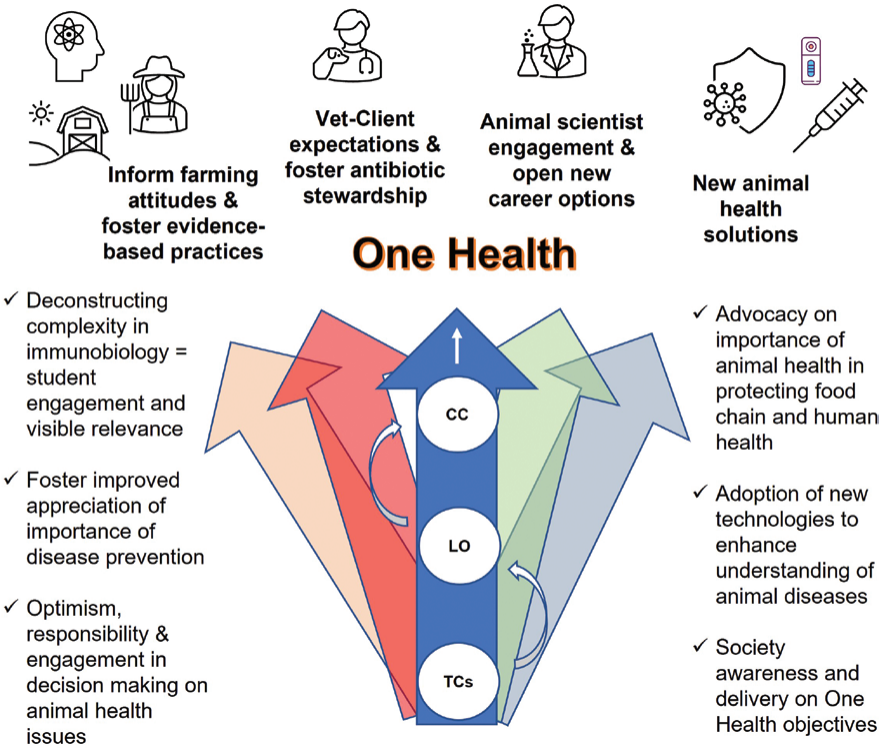
Immunobiology as a platform for One Health engagement. Consequential changes in expectations, behaviors, engagement, and practices of multiple stakeholders involved in animal health (and broader society) which will aid in the achievement of a One Health framework as a result of integration of Threshold Concepts (TCs) to achieve learning objectives (LO) within a connected curriculum (CC) for animal science.

An important aim is to equip these students with the skills to critically engage with research (Alpi and Hoggan, 2016) to inform their future decisions in terms of careers and business endeavors in the agricultural sector, specifically in terms of animal health. The pace of research is only going to grow and therefore a serious objective should be to expand the integration of research into the undergraduate animal science curriculum (Jones and Lerner, 2019). This is of particular relevance as a significant proportion of undergraduate animal science students have the ambition to progress their studies toward a future degree in veterinary science or medicine. Immunobiological literate animal science students will have better options in terms of engaging in immunology research as postgraduate students and research officers. Livestock immunology also has widespread relevance across biomedical fields. Farm animals have made sentinel contributions to immunological discoveries and continue to be used as models for human infections (Guzman and Montoya, 2018; Wagar et al., 2018). However, there are also particular dimensions to animal health research that require an appreciation of a very specific agricultural context (Ducrot et al., 2011). This highlights a valuable role for the prepared animal science graduates (Figure 3).

From an employment perspective, companies increasingly seek graduates with a comprehensive and global outlook, equipped to tackle the complexity of agri-food systems. It is in response to the magnitude of these challenges that all major pharmaceuticals and animal nutrition companies are expanding to embrace emerging innovative approaches in new drug development, vaccines, therapeutics, digital tools, and artificial intelligence amongst others (Ezanno et al., 2021). The Animal Health market size is expected to reach over USD 67 billion by 2026 representing a sizeable opportunity for future employment. The structural change happening across global agriculture will undoubtedly generate expanded and completely new areas in which animal science graduates could, if properly equipped, occupy leadership roles. This is reflected in the growth in the size and prospects for the pharmaceutical animal health sector. According to a 2020 report, agricultural technology has seen a huge growth in investment, with USD 6.7 billion invested in the last 5 yr alone (Deloitte and SCIO, 2020). New genomic sequencing technologies are revolutionizing our understanding of animal agriculture in general and infectious diseases in particular (Van Borm et al., 2015). However, the promise of these technologies has been recently regarded as limited by the availability of appropriate expertise and it suggested that the community of animal scientists help move from observational to hypothesis-driven research (Lippolis et al., 2019). If adequately resourced, the net effect will be to empower future animal science students to participate more fully in the pharmaceutical and technology sectors where their skill set is valuable but where they currently are underrepresented.

Traditionally, the animal science curriculum has an applied nature (Kauffman, 1992), of critical relevance to graduates who are practitioners and therefore the applied knowledge is of immediate application and consequence. The curriculum should maintain that core relevance but should also be responsive and flexible to expanding and emerging scientific areas (Erickson et al., 2020). By maintaining an understanding of the educational, experiential, and social facets of a programme, as well as programmatic outcomes, educators can more successfully prepare undergraduates in agriculture for the challenging futures that await them (Deslauriers et al., 2016). Employment prospects are therefore radically changing. It is the duty therefore of progressive third-level educational establishments to lead the way in pedagogical innovation to equip future generations of animal scientists with the knowledge and multidisciplinary skillsets to change farming practice and occupy leadership positions and delivering a sustainable future (Sørensen et al., 2021). As stated in a detailed analysis of the “Role of Animal Science Research in Food Security and Sustainability,” “meeting the challenges of sustainability over the next 40 yr will require more and different animal science research, extension, and education efforts” (National Research Council, 2015). This will require in-depth specialized knowledge and advancement of the basic sciences of animal health systems.

### Positive Societal Impacts of Transformative Learning in Immunobiology—a Portal to One Health

The widespread benefits of threshold concepts in immunobiology will extend beyond the graduates and have broader societal relevance as a result of the transformative education that occurs in the student but also that which permeates outwards through their practices and engagement with others (see Table 2). A call to collective action is now imperative at a societal level. This collective action involves the farmer and the veterinary health sector in partnership. The threshold concepts reflect the importance of programming for optimal animal health, way in advance of the onset of disease (Veit and Browning, 2021). Efforts to promote herd health and antibiotic stewardship initiatives on-farm will fail without improved literacy in immunobiology and active engagement by primary practitioners (Kramer et al., 2017; Liu et al., 2019). Evidence-based foundations from research and translated into practice will ultimately benefit all practitioners and allied health professionals (Figure 3).

Consumers too are increasingly concerned with the origin and quality of their food, and this extends to the welfare conditions of the animals being raised for food. Optimal animal welfare is contingent on minimizing animal disease, and therefore immunobiology has widespread relevance. Reconnecting society with the origin of its food (Ikerd, 2019) heralds an essential recognition for animal science ambassadors of the future. In addition, advocating a move toward preventative health implies proactive engagement and a commitment by farmers to raise livestock and produce food to the highest standards. This is a societal good and it is important that broader society recognize and reward the complexity of safe food systems. Transformative learning will therefore generate evidence-based advocates for the best practice in animal health. The active engagement of the public in animal health will also have many other knock-on benefits including maintaining societal support for research (Bottaro et al., 2021) and equip society to fight the spread of misinformation and to tackle vaccine hesitancy (Bottaro et al., 2021).

The One Health initiative is a global effort to harness collective efforts spanning animal and human health to restore sustainability and reverse the widespread damage to biodiversity, climate, and health (Destoumieux-Garzon et al., 2018). The challenges in animal health require specific expertise (Ducrot et al., 2011) and as a result, the modern farmer and future animal scientist have an increasingly relevant role to play in modern society (National Research Council, 2015). Furthermore, embedding threshold concepts in immunobiology is of direct relevance to the implementation of a curriculum adopting a One Health framework (Linder et al., 2020).

## CONCLUSION

A paradigm shift in animal health is required for overall food system sustainability (Perry et al., 2018) and so the time for disruptive innovation is now (Bloom and Cadarette, 2019; Shahpar et al., 2019). If the competences required to address potentially devastating zoonotic diseases are to be systematically developed, then pedagogies must deliberately foster them (Bedford et al., 2019). With over 570-million farms globally (Lowder et al., 2016), the power to effect positive change for system sustainability, food security, and human health lies herein. There is no doubt that animal production enterprises will continue to dramatically change (Tait-Burkard et al., 2018), it is, therefore, an exciting time to graduate as an animal scientist and opportunities exists to shape the future of the animal health industry (Tait-Burkard et al., 2018). Appreciating the expanding relevance of immunobiology will equip future generations of animal scientists to overcome issues currently impacting animal health, system sustainability, and enable active participation in our collective One Health agenda.
